# Reliability and Validity of the European Child Environment Questionnaire (ECEQ) in Children and Adolescents with Cerebral Palsy: Persian Version

**DOI:** 10.3390/children5040048

**Published:** 2018-04-09

**Authors:** Mahyar Salavati, Roshanak Vameghi, Seyed Ali Hosseini, Ahmad Saeedi, Masoud Gharib

**Affiliations:** 1 Department of Physiotherapy, University of Social Welfare and Rehabilitation Sciences, 1985713834 Tehran, Iran; Mahyarsalavati@gmail.com; 2 Pediatric Neurorehabilitation Research Center, University of Social Welfare and Rehabilitation Sciences, 1985713834 Tehran, Iran; R_vameghi@yahoo.com (R.V); alihosse@gmail.com (S.A.H.); 3 Department of Statistical Research and Information Technology, Institute for Research and Planning in Higher Education, 1985713834 Tehran, Iran; a_saidee@yahoo.com

**Keywords:** environment, child, cerebral palsy, questionnaire, validity

## Abstract

The aim of this study was to assess the reliability and validity of the Persian version of the European Child Environment Questionnaire (ECEQ) in the Iranian context. In total, 332 parents (20.2% fathers and 79.8% mothers) of children and adolescents with cerebral palsy (CP) with an average age of 12.33 years (min 7.08 to max 18.08) from three provinces in Iran participated in the study. The original version of the questionnaire was translated and back-translated. Confirmatory construct validity was assessed by factor analysis and reliability was evaluated by Cronbach’s alpha (*N* = 332) and after two weeks’ test–retest reliability (*n* = 51) using an intraclass correlation coefficient (ICC). Eleven questions were dropped as they did not fit well into domains in the Persian version (*p* > 0.05). Cronbach’s alpha and intraclass correlation coefficient in all domains and overall were acceptable (higher than 0.70) and significant (*p* > 0.05). The Persian version of the ECEQ is suitable for assessing the needs and availability of environmental factors and is reliable and valid for children with CP, as reported by their parents.

## 1. Introduction

Cerebral palsy (CP) is a prevalent motor disability in early childhood that more frequently affects posture and movement and causes activity limitations [1]. The motor disorder is generally associated with deficits in sensation, perception, cognition, behavior, and communication; seizures; and secondary problems such as deformity and contracture [2]. These restrictions and problems influence the participation of children with CP in different environments (contexts) such as the community, home, and school [3,4]. The type and severity of CP can affect participation, but some studies emphasize the role of the environment and the living place in the participation of children with cerebral palsy [5]. Hammal et al. found that there were differences in the participation of children with CP based on where they lived, regardless of the type and severity of their disability [6]. Especially in the case of children with CP in which most significant motor difficulties usually occur in the first seven years of life, an environment without barriers can generate positive results in the child’s community participation and higher levels of quality of life [7,8]. The International Classification of Functioning, Disability, and Health (ICF) considers disability to result from an interaction between a person’s intrinsic impairment and their physical, social, and attitudinal environment [9]. This is consistent with the social model of disability [10]. It is therefore of interest to develop measures that assess the availability to disabled people of the environmental features that they need. Based on the World Health Organization (WHO)’s ICF, environmental factors include (I) products and technology; (II) the natural environment and human-made changes to environment; (III) support and relationships; (IV) attitudes; and (V) service systems and policies [9]. Environmental factors can influence the “capacity” (what a child can do in an ideal environment) and the “performance” (what a child actually does in the environment in which s/he lives) of a child [11]. Different questionnaires have been developed to measure quantitatively the environmental needs and access rate of disabled adult persons [12]. Other questionnaires exist for disabled children in terms of the environment; however, either their main focus is mostly school settings [13] or they are developed for clinical assessment and evaluation and treatment planning purposes [14,15]. Yet, some other tools which were originally developed to measure the quality of the environment of adults have been subsequently adapted for children [12].

The European Child Environment Questionnaire (ECEQ), which was developed by a group studying children with CP living in Europe (SPARCLE, Study of Participation of Children with cerebral palsy Living in Europe.), is a convenient and comprehensive questionnaire produced to assess the environmental needs and accessibility of environmental factors for children with CP [12,16]. The ECEQ is a parent-report questionnaire with three domains (physical environment, social support, and attitude). The physical environment domain includes four sections (home, school, communication, and transport), the social support domain contains three sections (home, school, and communication), and the attitude domain consists of two sections: home and school [12]. Since this tool had not been evaluated for psychometric properties in the population of Iranian children with CP ever before, the aim of the present study was thus to assess the reliability and validity of the Persian version of the ECEQ in Iranian children and adolescents with cerebral palsy.

## 2. Materials and Methods

After obtaining permission from the head of the SPARCLE group for developing the Persian version of the ECEQ, the methodology introduced by the International Commission Tests [17,18,19] was adopted in order to ensure semantic, cultural, and conceptual integrity of the Persian version. Thus, the following process was followed: (1) two independent translators performed the Persian translation; (2) the translations were compared and agreed upon by the two translators, and eventually the preliminary version of the Persian questionnaire was achieved. Back-translation was carried out by a bilingual translator whose native language was English. At this point, the research team, as well as the expert panel, decided that the items in this questionnaire needed three-choice answers. Therefore, the previous answering and scoring system in which the “need” of each item was assessed and scored by two choices: “not needed = 0”and “needed = 1”; and “availability” of each item was assessed and scored by two other choices: “needed and available = 0” and “needed and not available = 1” [12,20], was changed to the following three-choice answers: “does not need = 3”, “needs and is available = 2”, and “needs but is not available = 1”. Finally, the head of the SPARCLE group, who was himself one of the developers of the ECEQ, was contacted and the final version of the Persian questionnaire was approved by him. In the next step, the face and content validity and cultural adaptability of the questionnaire was assessed by 10 parents of children with CP and five independent experts. For evaluating the validity and reliability of the questionnaire, a convenience sample of families recruited from private rehabilitation clinics in Tehran, Alborz, and Mazandaran provinces, who had children and adolescents with CP, were invited to this study. Families who could speak and read Persian (Farsi) fluently were included. Informed consent was acquired from participants. For assessing test–retest reliability, 51 parents were asked to complete the questionnaire again after two weeks. Confirmatory construct validity was assessed through factor analysis by AMOS software (SPSS Inc., Chicago, IL, USA) Reliability was assessed by examining internal consistency (using Cronbach’s alpha) and test–retest reliability using an intraclass correlation coefficient (ICC) by SPSS version 22 (SPSS Inc.). The research had ethical approval from the University of Social Welfare and Rehabilitation Sciences.

### Statistical Analysis

In order to determine the construct validity of the questionnaire, we followed the model proposed by Dickinson and Colver [12], who used confirmatory factor analysis by AMOS [21]. Factor loadings were estimated using generalized least squares. Validity of models was assessed by chi-square, the root mean square error of approximation (RMSEA), goodness-of-fit statistic (GFI), adjusted goodness-of-fit index (AGFI), and relative/normed chi-square (*χ*^2^/*df*) [22]. The chi-squared value is the traditional measure for evaluating overall model fit. A good model fit would provide an insignificant result at a 0.05 threshold [23]. While the chi-squared test retains its popularity as a fit statistic, there exist a number of severe limitations in its use. Due to the restrictiveness of the model chi-square, researchers have sought alternative indices to assess model fit such as the RMSEA, GFI, AGFI, and relative/normed chi-square (*χ*^2^/*df*) [22]. Recommendations for RMSEA cutoff points are different, but a range of between 0.05 and 0.10 is considered acceptable [24]. Values for the GFI and AGFI also range between 0 and 1, and it is generally accepted that values of 0.90 or greater indicate well-fitting models [25]. Recommendations for acceptable relative chi-squared values (*χ*^2^/*df*) range from as high as 5.0 [26] to as low as 2.0 [22].

To evaluate internal consistency and test–retest reliability, Cronbach’s alpha (*N* = 332) and ICC (*n* = 51), respectively, were assessed for each domain and the questionnaire overall. In all cases, the statistical significance was 0.05.

## 3. Results

During the face and content validity phase carried out by experts, the original 60 items of the questionnaire were maintained. However, item 32 (Does your child get specialized therapy services, such as: physical therapy, speech therapy, and occupational therapy?) was replaced by three questions separately addressing physical therapy, speech therapy, and occupational therapy, respectively. Thus, the number of items in the questionnaire finally reached 62 questions.

In total, 332 parents (20.2% fathers and 79.8% mothers) of children and adolescents with CP participated in the study. Of the children and adolescents,36.4% were girls and 63.6% were boys, with an average age of 12.33 (7.08 to 18.08) years. In order to assess the status of the motor function of the lower and upper extremities in these children, the Gross Motor Function Classification System (GMFCS) and Manual Ability Classification System (MACS)tests were performed for each child; the scoring distribution of which were 19.6% and 13.6% level I and 20.5% and 5.7% level V, respectively. The distribution of personal and sociodemographic characteristics of the participants (children) are shown in Table 1. In terms of the psychometric properties of the questionnaire, Cronbach’s alpha in all domains and overall was good (Cronbach’s alpha higher than 0.70) (Table 2). In total, Cronbach’s alpha was 0.914 and in the physical environment, social support, and attitude domains, it was 0.825, 0.849, and 0.736, respectively. Test–retest reliability using the ICC for the physical environment, social support, and attitude domains, as well as overall, were 0.978, 0.957, 0.719, and 0.979, respectively, with acceptable significance (*p* < 0.001) (Table 3).

In terms of the construct validity of the questionnaire, the results showed that the model chi-square suggested poor fit; on the other hand, the original domains of physical environment, social support, and attitude were not in good fit (*p* < 0.001) (Table 3). So, we assessed alternative indices for model fit such as the RMSEA, GFI, adjusted goodness-of-fit statistic (AGFI), and relative/normed chi-square (*χ*^2^/*df*) in domains. In the physical environment domain, RMSEA was 0.084, which is acceptable. AGFI and GFI were 0.788 and 0.825, respectively, which is acceptable. *χ*^2^/*df* was also acceptable (3.174) (Table 3). In the social support domain, AGFI and GFI were 0.730 and 0.780, respectively, which is acceptable. *χ*^2^/*df* was acceptable (4.438), but RMSEA was not acceptable (0.186) (Table 3). In the attitude domain, AGFI and GFI were acceptable, but RMSEA and *χ*^2^/*df* were not acceptable. In regard to outcomes, the models showed good fit in all three, but some questions did not have significant factor loadings (Table 3).

Table 3 shows how 332 parents answered the 62 questions of the questionnaire, as well as the dropped and included items, significance of each item, and model fit.

According to Table 3, at least half of participants said they needed the following items, but they were not available: enlarged rooms (50%), adapted toilet (60.5%), and modified kitchen (54.8%) at home; lifts at school (66.0%); ramps in public places (57.5%); adapted toilets in public places(81.0%); escalators in public places(57.5%); suitable doorways in public places(51.2%); ramps in public places (57.5%); smooth pavements in the town or village center (74.1%); accessible car parking (74.1%); adequate bus services (55.1%); accessible buses (57.5%). 

Also, in the social support section of the questionnaire, suitable leisure facilities (67.8%); receiving grants for home modifications (70.5%); access to information about financial benefits(83.7%); receiving grants for holidays (70.5%); receiving grants for equipment (54.2%); positive public attitude towards the child (59.6%); and existence of parent support groups in the area (72.9%) were reported to be needed, but were not available (Table 3). 

In the physical environment domain and the “community” section, items number 6 (factor loading = 0.083) and 7 (factor loading = 0.87) were not significant, and also, in the “transport” section, items 12 (factor loading = 0.038), 15 (factor loading = −0.535), and 16 (factor loading = −0.092) were not significant (*p* > 0.05) (Table 3), so five questions were dropped in this domain.

In the social support domain and the “home” section, items 25 (factor loading = 0.09) and 28 (factor loading = −0.27); and in the “community” section, items 32 (factor loading = 0.19) and 34 (factor loading = −0.08) were not significant (*p* > 0.05), so four questions were dropped in this domain (Table 3).

Finally, in the attitude domain and the “home” section, item 31 (factor loading = 0.03), and in the “school” section, item 55 (factor loading = 0.78), were not significant (Table 3), so two were questions dropped in this domain. Thus, 11 items were dropped from the original version of the questionnaire.

## 4. Discussion

In our study, we detected that according to parents’ reports, several of the CP children’s needs were not in included the physical environment domain, such as adapted toilets, lifts, suitable doorways; room in public places to move around; smooth pavements and accessible car parking in town. Dickinson and Colver [12] and Badiaetal [20] found similar results related to the physical environment domain. 

In our sample, there were zero cases of the “needs but is not available” choice of answers to question number 53; that is, “specialized therapy services (Occupational Therapy)”. This was expected, because all participants in this study used occupational therapy services, both in private and public centers.

In our study, the majority (67.8%) of parents reported that their children needed but did not have access to suitable leisure facilities. This is higher than what was reported by Dickinson and Colver [12], but lower than that reported by Badia [20]. These differences may be due to different socioeconomic factors related to the family or the society as well as to differences in the children’s characteristics. Longo et al. [27] believed that intelligence quotient level and GMFC were the most important factors for leisure participation in children and adolescents with CP.

In our study, parents reported their highest level of needs (whether available or not available) for adequate vehicles (96%) (question 11, which is in the physical environment domain); the responses to question 11 show that the need for having a suitable transportation system or providing special vehicles for children and adolescent with CP is high.

It is noteworthy that there were zero cases who reported “need and availability” for elevators at school (question 51). We think that due to safety issues, there are no elevators or lifts in any school in Iran. 

The answers to question number 40 show that the needs of parents for supporting groups are high. It seems that local public and private organizations should assist parents of children with CP in the formation of such support groups.

We know that causal indicators may be affected in this research; for example, a person who owns a personal car does not need accessible bus, taxi, and trains; or social support items may vary in some areas.

We think and hope that the findings of this study, especially the responses of parents to questions in each category, can increase the knowledge and awareness of communities, rehabilitation experts, rehabilitation managers, and policy makers regarding the needs of children with CP and their families, in order to provide more adapted physical surroundings, social support systems, and a positive-attitude environment, so as to enhance the quality of life and participation of children and adolescents with CP. 

Finally, our results have shown that based on parents’ response rate, the ECEQ was seemingly interesting to parents of children with CP. Also, following some refinement of the questionnaire, the ECEQ proved to be valid and reliable. However, according to factor analysis, the attitude domain seems to require more research, which may be due to the “latent trait” that is not directly subjective but affects responses to items, which was suggested and described by Dickinson and Colver [12] and Badia et al. [20].

Previous studies had not assessed the reliability of the ECEQ questionnaire by test–retest for determination of the ICC or by assessing Cronbach’s alpha coefficient. Future studies should investigate other psychometric properties of the ECEQ: Persian version. 

The limitation of this study is that the ECEQ, which is a parent-report questionnaire, may not have reflected the true needs of the child himself/herself. We suggest that other researchers develop a child-report version of this questionnaire.

## 5. Conclusions

The Persian version of the ECEQ questionnaire proved to be a valid and reliable tool for identifying the needs and availability of various environmental features for children with CP, as reported by their parents. Utilizing it can thus provide valuable information for policy-makers as well as clinicians for understanding and ultimately improving the environmental status of this group of children.

## Figures and Tables

**Table 1:** Personal and sociodemographic characteristics of children (*N* = 332).

Variable	Frequency (%)
**Sex**	
Boy	211 (63.6)
Girl	121 (36.4)
**Gross Motor Function Classification System (GMFCS)**	
Level I: walks and climbs stairs, without limitation	65 (19.6)
Level II: walks with limitations	66 (19.9)
Level III: walks with assistive devices	77 (23.2)
Level IV: unable to walk, limited self-mobility	56 (16.9)
Level V: unable to walk, severely limited self-mobility	68 (20.5)
**Manual Ability Classification System (MACS)**	
Level I: handles objects easily	45 (13.6)
Level II: handles objects with reduced quality and speed	131 (39.5)
Level III: handles objects with difficulty, needs help	77 (23.2)
Level IV: handles a limited selection of easily managed objects in adapted situations	59 (17.8)
Level V: does not handle objects.	19 (5.7)
**Intelligence Quotient (IQ) Levels**	
IQ > 70	176 (53)
IQ: 50–70	98 (29.5)
IQ < 50	58 (17.5)
**Type of Schooling**	
Regular	130 (39.2)
Special	202 (60.8)
**Reporting Parent**	
Father	67 (20.2)
Mother	265 (79.8)

**Table 2:** Cronbach’s alpha (*N* = 332) and internal consistency (*n* = 51) of the ECEQ subscales, and total.

ECEQ Subscales	Cronbach’s Alpha	ICC (95% CI)	*p*	*df*
Physical Environment	0.825	0.978 (0.962–0.988)	*p* < 0.001	50
Social Support	0.849	0.957 (0.925–0.976)		50
Attitudes	0.736	0.719 (0.509–0.840)		50
Total	0.914	0.979 (0.963–0.988)		50

ECEQ: European Child Environment Questionnaire, ICC: intraclass correlation coefficient, CI: confidence interval, *df*: degrees of freedom.

**Table 3:** Summary of responses and refinement of domain structure to ECEQ items by 332 families.

Dimension	Items Dropped from Domain	Factor Loading	Items Included in Domain	Model Fit					% Responders in Each Category		
				*χ*^2^	*df*	*p*	RMSEA	AGFI	Not Needed	Needed and Available	Needed and Not Available
**Physical environment**	6, 7, 12, 15, 16		1, 2, 3, 17, 18, 19, 47, 49, 50, 51, 52, 4, 5, 8, 9, 10, 11, 13, 14	787.215	248	*p* < 0.001	0.084	0.788			
**Home**											
1. Enlarged rooms at home		0.4	1, 2, 3, 17, 18, 19, 47						25.6	24.4	50
2. Adapted toilet at home		0.596							16	23.5	60.5
3. Modified kitchen at home		0.362							35.5	9.6	54.8
17. Walking aids		0.632							30.7	58.4	10.8
18. Hoists at home		0.66							56.6	2.1	41.3
19. Communication aids at home		0.481							78.9	3	18.1
47. Wheelchair or modified buggy		0.698							55.4	22.9	21.7
**School**			49, 50, 51, 52								
49. Ramps at school		0.671							31.6	38.9	29.5
50. Adapted toilets at school		0.615							28.6	50.3	21.1
51. Lifts at school		0.804							34	0	66
52. Communication aids at school		0.579							69.9	2.4	27.7
**Community**	6, 7		4, 5, 8, 9, 10								
4. Ramps in public places		0.659							31.3	11.1	57.5
5. Adapted toilets in public places		0.38							10.8	8.1	81
6. Lifts in public places		0.083 * n.s							16	44	40.1
7. Escalators in public places		0.87 * ^n.s.^							19.9	22.6	57.5
8.Suitable doorways in public places		0.536							16.6	32.2	51.2
9. Room in public places to move around		0.48							21.4	21.1	57.5
10. Smooth pavements in town or village centre		0.476							16.6	9.3	74.1
**Transport**	12, 15, 16		11, 13, 14								
11. Adequate vehicle		0.123							3.9	85.5	10.5
12. Accessible car parking		0.038 * ^n.s.^							13	13	74.1
13. Adequate bus service		−0.891							30.4	14.5	55.1
14. Accessible buses		−0.748							26.8	15.7	57.5
15. Accessible train services		−0.535 * ^n.s.^							53.9	18.1	28
16. Accessible taxis		−0.092 * ^n.s.^							43.7	29.5	26.8
**Social support**	25, 28, 32, 34		20, 21, 22, 23, 26, 27, 38, 39, 30, 48, 53, 59, 62, 24, 29, 33, 35, 36, 37, 40, 41, 44	914.173	206	*p* < 0.001	0.186	0.73			
**Home**	25, 28	*p* < 0.001	20, 21, 22, 23, 26, 27, 38, 39								
20. Receiving grants for equipment		0.352							25	20.8	54.2
21. Receiving grants for home modifications		0.67							21.1	8.4	70.5
22. Receiving grants for holidays		0.976							16.6	2.1	81.3
23. Access to information about financial benefits		0.967							15.4	0.9	83.7
25.Emotional support from family members living in home		0.09 * ^n.s.^								63.6	36.4
26. Emotional support from wider family/friends		0.176								77.7	22.3
27. Physical help from family members living in home		0.126							5.7	69.6	24.7
28. Physical help from wider family/friends		−0.27 * ^n.s.^							23.8	34.3	41.9
38. Helper or assistant at home		0.253							44	13	43.1
39. Family look after child for a few hours		0.145							25	40.7	34.3
**School**			30, 48, 53, 59, 62								
30. Teachers/doctors listen to your views		0.234								72	28
48. Child has school placement she/he needs		0.036								53.6	46.4
53. Special staff help child in school		0.789							34.9	41.9	23.2
59. Child receives physical help from teachers/therapists		0.559							22.6	67.5	9.9
62. Teachers have understanding of medical condition		0.349							25.9	60.5	13.6
**Community**	32, 34		24, 29, 33, 35, 36, 37, 40, 41, 44								
24. Suitable leisure facilities		0.229								32.2	67.8
29. Child receives physical help from people in public places		0.229							31	23.5	45.5
32. Specialized therapy services (Physical Therapy)		0.19 * ^n.s.^							64.8	15.1	20.2
33. Specialized therapy services (Speech Therapy)		0.798							35.5	49.1	15.4
34. Specialized therapy services (Occupational Therapy)		−0.08 * ^n.s.^							0	100	0
35. Health service staff coordinate work well		0.443								83.4	16.6
36. Social services coordinate work well		0.815							31.6	28.6	39.8
37. Child looked after elsewhere for few days		0.601							44	13	43.1
40. Existence of parent support groups in area		0.302							26.2	0.9	72.9
41. Counseling available		0.32							18.7	40.1	41.3
44. People in public places have positive attitude towards child		0.495								40.4	59.6
**Attitudes**	31, 55	42, 43, 45, 46, 54, 56, 57, 58, 60, 61		399.036	53	*p* < 0.001	0.14	0.782			
**Home**	31	42, 43, 45, 46									
31. Child allowed extra time at home		0.03 * ^n.s.^							14.2	73.8	12
42. Family members living in home have positive attitude towards child		0.623								90.4	9.6
43. Wider family and friends have positive attitude towards child		0.896								68.7	31.3
45. Child encouraged to reach potential by family members living in home		0.65								87.7	12.3
46. Child encouraged to reach potential from wider family/friends		0.852								61.7	38.3
**School**	55	54, 56, 57, 58, 60, 61									
54. Child allowed extra time at school		0.234							24.1	49.4	26.5
55. Child encouraged to reach potential from teachers/therapists		0.78 * ^n.s.^								78.3	21.7
56. Child encouraged to reach potential from classmates		0.567								83.4	16.6
57. Child receives emotional support from teachers/therapists		0.438								88.6	11.4
58. Child receives emotional support from classmates		0.993								75	25
60. Teachers/therapists have positive attitude towards child		0.33								72.3	10.8
61. Classmates have positive attitude towards child		0.671								72.3	27.7

* *p* > 0.05, ^n.s.^: not significant, RMSEA: root mean square error of approximation; AGFI: adjusted goodness-of-fit statistic.
